# Supplementary material for: Oral microbiome alterations after cancer treatment: a scoping review and analysis
Source: Med Oncol. 2025 Aug 19;42(10):432. doi: 10.1007/s12032-025-02998-6 (PMC12364993; doi:10.1007/s12032-025-02998-6)
Supplement: Supplementary file 1 — Supplementary file1 (DOCX 99 KB) [file 12032_2025_2998_MOESM1_ESM.docx]

**Oral Microbiome Alterations After Cancer Treatment: A Scoping Review and Analysis**

# SUPPLEMENTAL MATERIAL

## Supplemental Table 1: Search Results

Database: PubMed/MEDLINE

Platform: US National Library of Medicine

Limits Used: Publication date: January 1, 1990-date of searches; Article types; Human studies

Note: [tiab] = title and abstract field; [MeSH] = Medical Subject Heading field; [ptyp] = publication type field

| **Query Number** | **Concept** | **Search Strategy** |
| --- | --- | --- |
| **#1** | Cancer Therapy Modality | ("Hematopoietic Stem Cell Transpl*"[Mesh] OR “allogenic stem cell transplant*”[tiab] OR “bone marrow transplant*”[tiab] OR “cancer chemotherapy agent*”[tiab] OR “cancer chemotherapy drug*”[tiab] OR “cancer therap*”[tiab] OR chemotherap*[tiab] OR “haematopoietic stem cell transplant*”[tiab] OR “immune block*”[tiab] OR “immune checkpoint inhibitor*”[tiab] OR immunotherap*[tiab] OR “radiation therap*”[tiab] OR radioimmunotherap*[tiab] OR “targeted cancer therap*”[tiab] OR “targeted therap*”[tiab] OR “targeted molecular therap*”[tiab] OR “targeted immunotherap*”[tiab]) |
| **#2** | Oral Microbiome | (“dental plaque”[tiab] OR “mouth bacteria”[tiab] OR “mouth candida”[tiab] OR “mouth candidiasis”[tiab] OR “mouth flora”[tiab] OR “oral bacteria”[tiab] OR “oral bacterium”[tiab] OR “oral bacterial flora”[tiab] OR “oral bacterial profile*”[tiab] OR “oral biofilm*”[tiab] OR “oral candida”[tiab] OR “oral candidiasis”[tiab] OR “oral cavity flora”[tiab] OR “oral dysbiosis”[tiab] OR “oral flora”[tiab] OR “oral fungus”[tiab] OR “oral fungal infection*”[tiab] OR “oral microbe*”[tiab] OR “oral microbial”[tiab] OR “oral microbial flora”[tiab] OR “oral microbiome”[tiab] OR “oral microbiota”[tiab] OR “oral microflora”[tiab] OR “oral microorganism*”[tiab] OR “salivary bacterial profile*”[tiab] OR “sub gingival bacteria”[tiab] OR “subgingival bacteria”[tiab] OR “supra gingival bacteria”[tiab] OR “supragingival bacteria”[tiab] OR “throat flora”[tiab] OR thrush[tiab] OR “salivary microbiome”[tiab]) |
| **#3** |  | #1 AND #2 |
| **#4** | Limits Applied: Publication date, Language, Article type, Exclude animal studies | #3 AND (("1990/01/01"[Date - Publication] : "3000"[Date - Publication])) AND English[lang] AND (case reports[ptyp] OR classical article[ptyp] OR clinical conference[ptyp] OR clinical study[ptyp] OR clinical trial[ptyp] OR comparative study[ptyp] OR controlled clinical trial[ptyp] OR evaluation study[ptyp] OR historical article[ptyp] OR introductory journal article[ptyp] OR journal article[ptyp] OR meta-analysis[ptyp] OR multicenter study[ptyp] OR observational study[ptyp] OR pragmatic clinical trial[ptyp] OR  randomized controlled trial[ptyp] OR review[ptyp] OR “systematic review”[ptyp] OR validation study[ptyp] OR letter[ptyp] OR editorial[ptyp]) NOT (animals[mesh] NOT humans[mesh]) |

## Supplemental Table 2: PRISMA-ScR Checklist

Preferred Reporting Items for Systematic reviews and Meta-Analyses extension for Scoping Reviews (PRISMA-ScR) Checklist:

| **SECTION** | **ITEM** | **PRISMA-ScR CHECKLIST ITEM** | **REPORTED ON PAGE #** |
| --- | --- | --- | --- |
| **TITLE** | | | |
| Title | 1 | Identify the report as a scoping review. | Click here to enter text. |
| **ABSTRACT** | | | |
| Structured summary | 2 | Provide a structured summary that includes (as applicable): background, objectives, eligibility criteria, sources of evidence, charting methods, results, and conclusions that relate to the review questions and objectives. | Click here to enter text. |
| **INTRODUCTION** | | | |
| Rationale | 3 | Describe the rationale for the review in the context of what is already known. Explain why the review questions/objectives lend themselves to a scoping review approach. | Click here to enter text. |
| Objectives | 4 | Provide an explicit statement of the questions and objectives being addressed with reference to their key elements (e.g., population or participants, concepts, and context) or other relevant key elements used to conceptualize the review questions and/or objectives. | Click here to enter text. |
| **METHODS** | | | |
| Protocol and registration | 5 | Indicate whether a review protocol exists; state if and where it can be accessed (e.g., a Web address); and if available, provide registration information, including the registration number. | Click here to enter text. |
| Eligibility criteria | 6 | Specify characteristics of the sources of evidence used as eligibility criteria (e.g., years considered, language, and publication status), and provide a rationale. | Click here to enter text. |
| Information sources* | 7 | Describe all information sources in the search (e.g., databases with dates of coverage and contact with authors to identify additional sources), as well as the date the most recent search was executed. | Click here to enter text. |
| Search | 8 | Present the full electronic search strategy for at least 1 database, including any limits used, such that it could be repeated. | Click here to enter text. |
| Selection of sources of evidence† | 9 | State the process for selecting sources of evidence (i.e., screening and eligibility) included in the scoping review. | Click here to enter text. |
| Data charting process‡ | 10 | Describe the methods of charting data from the included sources of evidence (e.g., calibrated forms or forms that have been tested by the team before their use, and whether data charting was done independently or in duplicate) and any processes for obtaining and confirming data from investigators. | Click here to enter text. |
| Data items | 11 | List and define all variables for which data were sought and any assumptions and simplifications made. | Click here to enter text. |
| Critical appraisal of individual sources of evidence§ | 12 | If done, provide a rationale for conducting a critical appraisal of included sources of evidence; describe the methods used and how this information was used in any data synthesis (if appropriate). | Click here to enter text. |
| Synthesis of results | 13 | Describe the methods of handling and summarizing the data that were charted. | Click here to enter text. |
| **RESULTS** | | | |
| Selection of sources of evidence | 14 | Give numbers of sources of evidence screened, assessed for eligibility, and included in the review, with reasons for exclusions at each stage, ideally using a flow diagram. | Click here to enter text. |
| Characteristics of sources of evidence | 15 | For each source of evidence, present characteristics for which data were charted and provide the citations. | Click here to enter text. |
| Critical appraisal within sources of evidence | 16 | If done, present data on critical appraisal of included sources of evidence (see item 12). | Click here to enter text. |
| Results of individual sources of evidence | 17 | For each included source of evidence, present the relevant data that were charted that relate to the review questions and objectives. | Click here to enter text. |
| Synthesis of results | 18 | Summarize and/or present the charting results as they relate to the review questions and objectives. | Click here to enter text. |
| **DISCUSSION** | | | |
| Summary of evidence | 19 | Summarize the main results (including an overview of concepts, themes, and types of evidence available), link to the review questions and objectives, and consider the relevance to key groups. | Click here to enter text. |
| Limitations | 20 | Discuss the limitations of the scoping review process. | Click here to enter text. |
| Conclusions | 21 | Provide a general interpretation of the results with respect to the review questions and objectives, as well as potential implications and/or next steps. | Click here to enter text. |
| **FUNDING** | | | |
| Funding | 22 | Describe sources of funding for the included sources of evidence, as well as sources of funding for the scoping review. Describe the role of the funders of the scoping review. | Click here to enter text. |

JBI = Joanna Briggs Institute; PRISMA-ScR = Preferred Reporting Items for Systematic reviews and Meta-Analyses extension for Scoping Reviews.

* Where *sources of evidence* (see second footnote) are compiled from, such as bibliographic databases, social media platforms, and Web sites.

† A more inclusive/heterogeneous term used to account for the different types of evidence or data sources (e.g., quantitative and/or qualitative research, expert opinion, and policy documents) that may be eligible in a scoping review as opposed to only studies. This is not to be confused with *information sources* (see first footnote).

‡ The frameworks by Arksey and O’Malley (6) and Levac and colleagues (7) and the JBI guidance (4, 5) refer to the process of data extraction in a scoping review as data charting*.*

§ The process of systematically examining research evidence to assess its validity, results, and relevance before using it to inform a decision. This term is used for items 12 and 19 instead of "risk of bias" (which is more applicable to systematic reviews of interventions) to include and acknowledge the various sources of evidence that may be used in a scoping review (e.g., quantitative and/or qualitative research, expert opinion, and policy document).

*From:* Tricco AC, Lillie E, Zarin W, O'Brien KK, Colquhoun H, Levac D, et al. PRISMA Extension for Scoping Reviews (PRISMAScR): Checklist and Explanation. Ann Intern Med. 2018;169:467–473. [doi: 10.7326/M18-0850](http://annals.org/aim/fullarticle/2700389/prisma-extension-scoping-reviews-prisma-scr-checklist-explanation).

## Supplemental Table 3: Article Information

| Title | Year | First Author | Source |
| --- | --- | --- | --- |
| Chemotherapy-induced oral mucositis is associated with detrimental bacterial dysbiosis | 2019 | Hong | Microbiome |
| Alterations of the Oral Microbiome and Cumulative Carbapenem Exposure Are Associated with Stenotrophomonas maltophilia Infection in Patients with Acute Myeloid Leukemia Receiving Chemotherapy | 2020 | Aitken | Clinical Infectious Diseases |
| Microflora analysis in the postchemotherapy patients of oral cancer | 2019 | Singh | National Journal of Maxillofacial Surgery |
| Prospective study of the long-term change of the oral flora after radiation therapy | 2005 | Al-Nawas | Supportive Care in Cancer |
| Oral microbiota associated with hyposalivation of different origins | 2003 | Almstahl | Oral Microbiology and Immunology |
| Incidence and spectrum of yeast species isolated from the oral cavity of Iranian patients suffering from hematological malignancies | 2019 | Arastehfar | Journal of Oral Microbiology |
| Alterations in Oral Microflora and Pathogenesis of Acute Oral Infections during Remission-Induction Therapy in Patients with Acute Myeloid Leukaemia | 2009 | Bergmann | Scandinavian Journal of Infectious Diseases |
| Prospective Evaluation of HSV, Candida spp., and Oral Bacteria on the Severity of Oral Mucositis in Pediatric Acute Lymphoblastic Leukemia | 2011 | Holanda de Mendonca | Supportive Care in Cancer |
| Distinct shifts in the oral microbiota are associated with the progression and aggravation of mucositis during radiotherapy | 2018 | Hou | Radiotherapy and Oncology |
| Dental Biofilm Microbiota Dysbiosis Is Associated with the Risk of Acute Graft-Versus-Host Disease After Allogeneic Hematopoietic Stem Cell Transplantation | 2021 | Heidrich | Frontiers in Immunology |
| The Oral Carriage of Candida in Oral Cancer Patients of Indian Origin Undergoing Radiotherapy and/or Chemotherapy | 2016 | Jain | Journal of Clinical and Diagnostic Research |
| Oral mucosal lesions, microbial changes, and taste disturbances induced by adjuvant chemotherapy in breast cancer patients | 2008 | Jensen | Oral Medicine (Oral Surgery, Oral Medicine, Oral Pathology, Oral Radiology, and Endodontology) |
| Oral bacteria and yeasts in relationship to oral ulcerations in hematopoietic stem cell transplant recipients | 2012 | Laheij | Supportive Care in Cancer |
| Oral Mucositis in Pediatric Acute Lymphoblastic Leukemia Patients: Evaluation of Microbiological and Hematological Factors | 2015 | Holanda de Mendonca | Pediatric Hematology and Oncology |
| Unusual oral mucosal microbiota after hematopoietic cell transplantation with glycopeptide antibiotics: potential association with pathophysiology of oral mucositis | 2018 | Muro | Folia Microbiologica |
| Commensal oral microbiota impacts ulcerative oral mucositis clinical course in allogeneic stem cell transplant recipients | 2022 | Bruno | Scientific Reports |
| Oral microorganisms and bloodstream infection in allogeneic hematopoietic stem cell transplantation | 2021 | Ohbayashi | Clinical Oral Investigations |
| Prevalence and susceptibility profile of Candida spp. isolated from patients in cancer therapy | 2020 | Castro de Souza e Silva | Archives of Oral Biology |
| An analysis of oral microbial flora by T-RFLP in patients undergoing hematopoietic stem cell transplantation | 2020 | Takahashi | International Journal of Hematology |
| Patterns of salivary microbiota injury and oral mucositis in recipients of allogeneic hematopoietic stem cell transplantation | 2020 | Shouval | Blood Advances |
| Evaluation of Alteration in Oral Microbial Flora Pre- and Postradiation Therapy in Patients with Head and Neck Cancer | 2020 | Priya | Journal of Pharmacy and Bioallied Sciences |
| Oral Microbiome and Onset of Oral Mucositis in Patients with Squamous Cell Carcinoma of the Head and Neck | 2020 | Reyes-Gibby | Cancer |
| Caries-associated oral microbiome in head and neck cancer radiation patients: a longitudinal study | 2019 | Mougeot | Journal of Oral Microbiology |
| Oral microbial profile in oral cancer patients before and after radiation therapy in a cancer care center – A prospective study | 2020 | Anjali | Journal of Oral and Maxillofacial Pathology |
| Disrupted tongue microbiota and detection of nonindigenous bacteria on the day of allogeneic hematopoietic stem cell transplantation | 2020 | Oku | PLOS Pathogens |
| Microbial changes in relation to oral mucositis in autologous hematopoietic stem cell transplantation recipients | 2019 | Laheij | Scientific Reports |
| Characterization of oral and gut microbiome temporal variability in hospitalized cancer patients | 2017 | Galloway-Peña | Genome Medicine |
| Salivary microbiome and oral mucositis in HSCT recipients: A pilot study | 2023 | Kwiatkowski | Oral Diseases |
| The recovery of intestinal barrier function and changes in oral microbiota after radiation therapy injury | 2024 | Wang | Frontiers in Cellular and Infection Microbiology |
| Oral and Gut Microbiome Alterations in Oral Chronic GVHD Disease: Results from Close Assessment and Testing for Chronic GVHD (CATCH Study) | 2024 | Rashidi | Clinical Cancer Research |
| Association between oral and fecal microbiome dysbiosis and treatment complications in pediatric patients undergoing allogeneic hematopoietic stem cell transplantation | 2024 | Faraci | Scientific Reports |
| Herpesviruses in the oral cavity of patients subjected to allogeneic hematopoietic stem cell transplantation and its relationship with oral mucositis | 2020 | Miranda-Silva | Clinical Oral Investigations |
| Improving Oral Health and Modulating the Oral Microbiome to Reduce Bloodstream Infections from Oral Organisms in Pediatric and Young Adult Hematopoietic Stem Cell Transplantation Recipients: A Randomized Controlled Trial | 2020 | Badia | Biology of Blood and Marrow Transplantation |
| Integrated Analysis of Clinical and Microbiome Risk Factors Associated with the Development of Oral Candidiasis during Cancer Chemotherapy | 2019 | Diaz | Journal of Fungi |
| Opportunistic Microorganisms in Oral Cavity According to Treatment Status in Head and Neck Cancer Patients | 2017 | Soni | Journal of Clinical and Diagnostic Research |
| Influence of Oral Anaerobic Bacteria on Hematopoietic Stem Cell Transplantation Patients: Oral Mucositis and General Condition | 2017 | Osakabe | Transplantation Proceedings |
| Capnocytophaga in the dental plaque of immunocompromised children with cancer | 2006 | Sixou | International Journal of Paediatric Dentistry |
| Oral Health, Caries Risk Profiles, and Oral Microbiome of Pediatric Patients with Leukemia Submitted to Chemotherapy | 2021 | Wang | BioMed Research International |
| Head and neck intensity modulated radiation therapy leads to an increase of opportunistic oral pathogens | 2016 | Schuurhuis | Oral Oncology |
| Microflora in oral ecosystems and salivary secretion rates – A 3-year follow-up after radiation therapy to the head and neck region | 2015 | Almstahl | Archives of Oral Biology |
| Bacteria and Candida yeasts in inflammations of the oral mucosa in children with secondary immunodeficiency | 2012 | Olczak-Kowalczyk | Journal of Oral Pathology & Medicine |
| Changes in microflora in dental plaque from cancer patients undergoing chemotherapy and the relationship of these changes with mucositis: A pilot study | 2014 | Vozza | Medicina Oral, Patologia Oral y Cirugia Bucal |
| The Potential Effect of Oral Microbiota in the Prediction of Mucositis During Radiotherapy for Nasopharyngeal Carcinoma | 2017 | Zhu | EBioMedicine |
| Oral bacterial community dynamics in paediatric patients with malignancies in relation to chemotherapy-related oral mucositis: a prospective study | 2013 | Ye | Clinical Microbiology and Infection |
| Molecular methodology to assess the impact of cancer chemotherapy on the oral bacterial flora: a pilot study | 2009 | Napenas | Oral Surgery, Oral Medicine, Oral Pathology, Oral Radiology, and Endodontics |
| Changes of saliva microbiota in nasopharyngeal carcinoma patients under chemoradiation therapy | 2013 | Xu | Archives of Oral Biology |
| Fluconazole Susceptibility and Genotypic Heterogeneity of Oral Candida albicans Colonies from the Patients with Cancer Receiving Chemotherapy in China | 2009 | Sun | International Journal of Oral Science |
| An Assessment of the Number of Cariogenic Bacteria in the Saliva of Children with Chemotherapy-Induced Neutropenia | 2016 | Olszekska | Advances in Clinical and Experimental Medicine |
| Mucositis Reduction By Selective Elimination of Oral Flora in Irradiated Cancers of the Head and Neck: A Placebo-Controlled Double-Blinded Randomized Study | 2001 | Wijers | International Journal of Radiation Oncology, Biology, Physics |
| Determination of Oral Microflora in Irradiated Ocular Deformed Children | 2006 | Srithavaj | The Southeast Asian Journal of Tropical Medicine and Public Health |
| Effect of granulocytopenia on oral microbial relationships in patients with acute leukemia | 1990 | Peterson | Oral Surgery, Oral Medicine, Oral Pathology |
| The correlation between the numbers of some bacteria in human oral cavity and blood neutrophil count | 1998 | Majda-Stanislawska | FEMS Immunology and Medical Microbiology |
| Characterization of Oral Microbiota Following Chemotherapy in Patients With Hematopoietic Malignancies | 2023 | Omori | Integrative Cancer Therapies |
| Gut microbiota diversity and specific composition during immunotherapy in responders with non-small cell lung cancer | 2022 | Shoji | Frontiers in Molecular Biosciences |
| The Effect of Intensity-Modulated Radiotherapy to the Head and Neck Region on the Oral Innate Immune Response and Oral Microbiome: A Prospective Cohort Study of Head and Neck Tumour Patients | 2022 | Mojdami | International Journal of Molecular Sciences |
| A prospective study of dysgeusia and related symptoms in patients with multiple myeloma after autologous hematopoietic cell transplantation | 2023 | Scordo | Cancer |
| Chemotherapy-associated oral microbiome changes in breast cancer patients | 2022 | Klymiuk | Frontiers in Oncology |
| Oral and Stool Microbiome Coalescence and Its Association With Antibiotic Exposure in Acute Leukemia Patients | 2022 | Franklin | Frontiers in Cellular and Infection Microbiology |
| Evaluation of Changes to the Oral Microbiome Based on 16S rRNA Sequencing among Children Treated for Cancer | 2021 | Proc | Cancers |
| Molecular identification and antifungal susceptibility testing of Candida species isolated from oral lesions in patients with head and neck cancer undergoing radiotherapy | 2021 | Kermani | Current Medical Mycology |
| Disruption of the oral microbiota is associated with a higher risk of relapse after allogeneic hematopoietic stem cell transplantation | 2021 | Campos de Molla | Scientific Reports |
| Microflora in oral ecosystems in subjects with radiation induced hyposalivation | 2008 | Almstahl | Oral Diseases |

# REFERENCES
